# Athermal domain-wall creep near a ferroelectric quantum critical point

**DOI:** 10.1038/ncomms10675

**Published:** 2016-02-16

**Authors:** Fumitaka Kagawa, Nao Minami, Sachio Horiuchi, Yoshinori Tokura

**Affiliations:** 1 RIKEN Center for Emergent Matter Science (CEMS), Wako 351-0198, Japan; 2 Department of Applied Physics, The University of Tokyo, Tokyo 113-8656, Japan; 3 National Institute of Advanced Industrial Science and Technology (AIST), AIST Tsukuba Central 5, 1-1-1 Higashi, Tsukuba 305-8565, Japan

## Abstract

Ferroelectric domain walls are typically stationary because of the presence of a pinning potential. Nevertheless, thermally activated, irreversible creep motion can occur under a moderate electric field, thereby underlying rewritable and non-volatile memory applications. Conversely, as the temperature decreases, the occurrence of creep motion becomes less likely and eventually impossible under realistic electric-field magnitudes. Here we show that such frozen ferroelectric domain walls recover their mobility under the influence of quantum fluctuations. Nonlinear permittivity and polarization-retention measurements of an organic charge-transfer complex reveal that ferroelectric domain-wall creep occurs via an athermal process when the system is tuned close to a pressure-driven ferroelectric quantum critical point. Despite the heavy masses of material building blocks such as molecules, the estimated effective mass of the domain wall is comparable to the proton mass, indicating the realization of a ferroelectric domain wall with a quantum-particle nature near the quantum critical point.

## Abstract (web summary)

At very low temperature, particle-like objects such as atoms and material phase boundaries become less able to move due to thermally inactivity. Here, Kagawa *et al*. show that ferroelectric domain walls gain energy for creep motion due to quantum fluctuations at low temperature.

## Graphical abstract

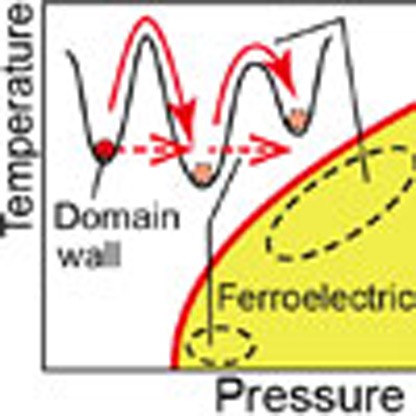

The nonequilibrium creep dynamics of elastic objects under small external forces is often described in reference to a phenomenological, multidimensional potential landscape with multiple minima. Typical examples include isolated/lattice-formed flux lines in superconductors under electric currents^1^, charge-/spin-density waves under electric currents^2^,^3^ and ferroelectric/ferromagnetic domain walls under electric/magnetic fields^4^,^5^,^6^,^7^,^8^,^9^,^10^,^11^,^12^,^13^,^14^,^15^,^16^. From a phenomenological perspective, when a moderate external force is applied, the system begins to travel through this multidimensional potential landscape by successively overcoming the potential barriers separating distinct metastable states, resulting in creep motion in real space. This creep motion is induced by thermal activation, or thermal fluctuations. An experimentally elusive question is how the creep kinetics would be affected when quantum fluctuations develop while thermal fluctuations are suppressed to the greatest possible extent.

In this work, to address this question, we targeted a recently discovered organic ferroelectric charge-transfer complex, TTF-QBr_2_I_2_ (ref. 17), where TTF and QBr_2_I_2_ denote tetrathiafulvalene and symmetrically diiodo-substituted quinone, respectively. A combination of nonlinear permittivity and polarization-retention measurements reveals that the domain-wall creep motion manifests athermal, quantum-mechanical behaviour as a result of quantum fluctuations developing towards a ferroelectric quantum critical point (QCP). The estimated effective mass of the ferroelectric domain wall is found to be much lighter than the mass of the material building blocks, which are molecules in this case, and this situation is reminiscent of the electrically mobile bond solitons in conducting polymers, such as polyacetylene.

## Results

### Phase diagram

**Figure 1:**
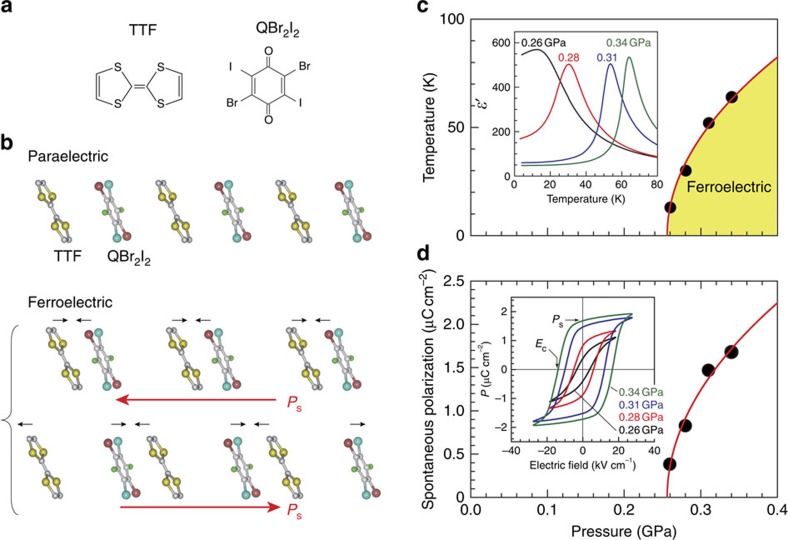
Quantum fluctuations developing near the ferroelectric quantum critical point in TTF-QBr_2_I_2_. (**a**) The chemical forms of the TTF and QBr_2_I_2_ molecules. TTF and QBr_2_I_2_ denote tetrathiafulvalene and symmetrically diiodo-substituted quinone, respectively. (**b**) Schematic diagrams of the structural changes occurring upon the ferroelectric transition. In the ferroelectric phase, the TTF and QBr_2_I_2_ molecules are dimerized, and the small arrows indicate the shifts in the molecules that occur upon dimerization. Two dimerization patterns are possible, and they are degenerate in energy. The red arrows indicate the direction of the spontaneous electric polarizations, *P*_s_. (**c**) The pressure–temperature phase diagram. The inset shows the temperature dependence of the permittivity (measured at 100 kHz) at the pressures investigated in this study. The ferroelectric transition temperatures can be determined from the permittivity peaks. (**d**) The pressure dependence of the spontaneous polarization at the lowest temperature. The inset shows the pressure variations in the polarization hysteresis loop measured at the lowest temperature: *T*/*T*_c_=0.1 for 0.28; 0.31 and 0.34 GPa; and *T*/*T*_c_=0.3 for 0.26 GPa.

TTF-QBr_2_I_2_ consists of alternating stacking of TTF and QBr_2_I_2_ with a uniform spacing, and it is paraelectric at ambient pressure down to the lowest temperature (Fig. 1a,b). Nevertheless, there exists a second-order ferroelectric transition accompanied by a QCP at a moderate pressure^17^, *p*_c_≈0.25–0.26 GPa (Fig. 1c), and above that pressure, the dimerization of TTF and QBr_2_I_2_ occurs and results in a ferroelectric polarization oriented approximately along the stacking direction (Fig. 1b). TTF-QBr_2_I_2_ thus provides an ideal platform to explore the kinetics of ferroelectric domain walls under systematically controlled quantum fluctuations. In fact, when the pressure approaches *p*_c_ from above, the ferroelectric transition temperature *T*_c_ continuously decreases to zero as *T*_c_∼(*p*−*p*_c_)^0.5^ (Fig. 1c), consistently with previously reported results^17^; moreover, the spontaneous polarization *P*_s_ at the lowest temperature decreases as *P*_s_∼(*p*−*p*_c_)^0.5^ (Fig. 1d). The square-root decreases in *T*_c_ and *P*_s_ are consistent with theoretical predictions that consider quantum fluctuation effects^18^,^19^, indicating that the quantum fluctuations evolve towards the pressure-driven ferroelectric QCP and are, therefore, tunable.

### Nonlinear permittivity arising from domain-wall creep

**Figure 2:**
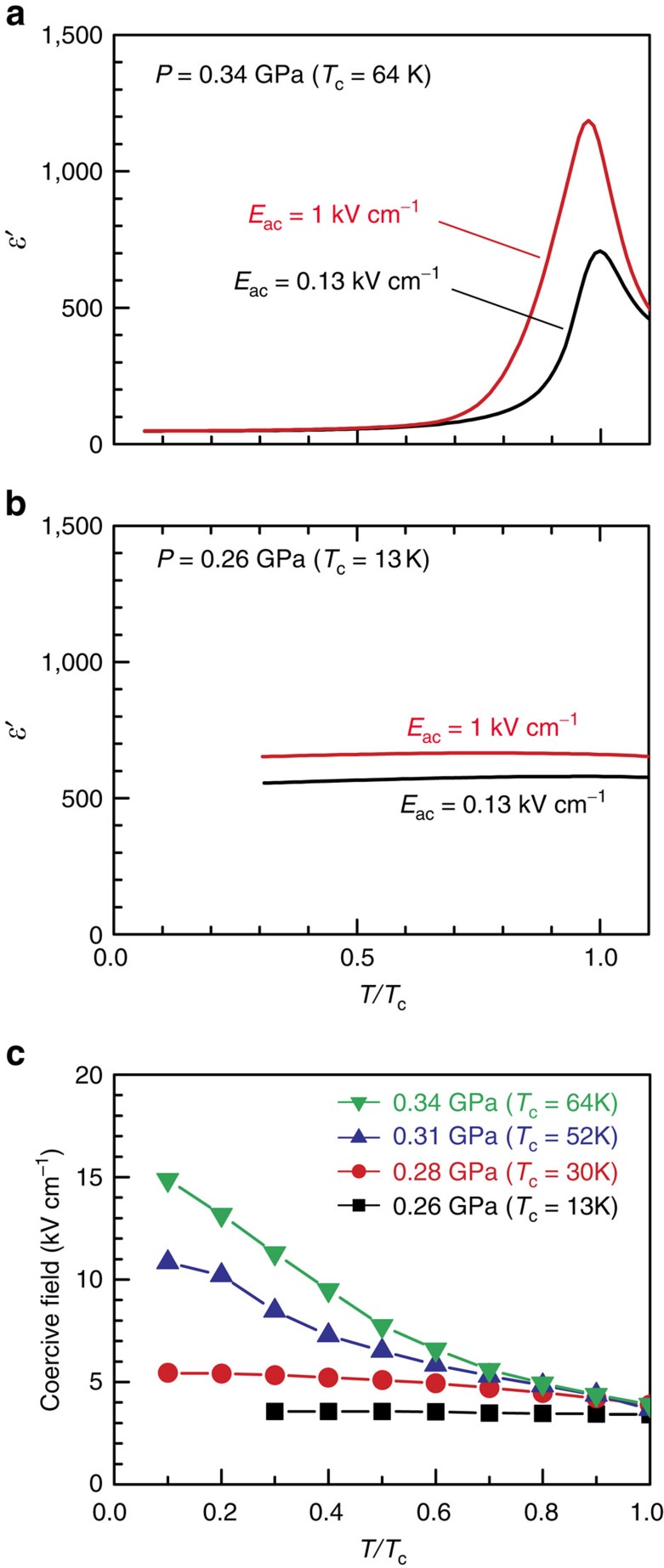
Probing the ferroelectric domain-wall creep via dielectric properties. (**a**,**b**) The permittivity-temperature profiles measured under a.c. electric fields of different magnitudes: at 0.34 GPa (*T*_c_≈64 K) (**a**) and at 0.26 GPa (*T*_c_≈13 K) (**b**), the latter of which is close to the ferroelectric QCP. (**c**) The temperature dependence of the coercive electric field at different pressures. In **a**–**c** the temperatures are represented in the form of reduced temperatures, *T*/*T*_c_.

In pursuing the nontrivial domain-wall dynamics arising from quantum fluctuations, we first focus on the domain-wall creep under an a.c. electric field, *E*_ac_, using permittivity measurements as a sensitive probe (for the schematics of the domain-wall structure, see Supplementary Fig. 1). Figure 2a displays the permittivity-temperature (*ɛ*′−*T*) profile measured at 0.34 GPa, which is relatively far from the ferroelectric QCP (≈0.25–0.26 GPa) and hence, yields a high *T*_c_ of ∼64 K (regarding possible ambiguity of *T*_c_, see Supplementary Note 1). We note that near *T*_c_, the *ɛ′* measured at *E*_ac_≈1 kV cm^–1^ exhibits larger values than that measured at *E*_ac_≈0.13 kV cm^–1^; that is, the permittivity exhibits highly nonlinear characteristics. Given that domain-wall creep is innately a nonlinear response to an external electric field^4^,^6^,^7^,^9^, the origin of this nonlinear permittivity is attributable to the domain-wall creep^8^ occurring under the application of a field of *E*_ac_≈1 kV cm^–1^ (the same conclusion can also be drawn from the frequency dependence of the permittivity; see Supplementary Fig. 2). For *T*/*T*_c_<0.6, the nonlinear permittivity becomes vanishingly small, thus highlighting the vital role of thermal fluctuations in the domain-wall creep at 0.34 GPa. By contrast, when the pressure is decreased to 0.26 GPa, which is near *p*_c_ and hence yields a low *T*_c_ (≈13 K), nonlinear permittivity is observed in a broad *T*/*T*_c_ range, and its magnitude depends only weakly on temperature (Fig. 2b), implying that the domain-wall creep at 0.26 GPa is an athermal process.

The possible crossover from thermal to athermal domain-wall creep is further supported by the temperature dependence of the coercive electric field, *E*_c_, in the polarization hysteresis loop (Fig. 2c); although at 0.34 GPa, *E*_c_ increases towards lower temperatures, it exhibits less significant variation and eventually becomes nearly temperature-invariant as the pressure approaches *p*_c_ (≈0.25–0.26 GPa). Because the polarization reversal process is generally accompanied by domain-wall creep, the pressure variation of the *E*_c_-*T* profile again suggests that although the domain-wall creep at 0.34 GPa is facilitated by thermal fluctuations, this is not the case at pressures near the ferroelectric QCP.

### Polarization decay

**Figure 3:**
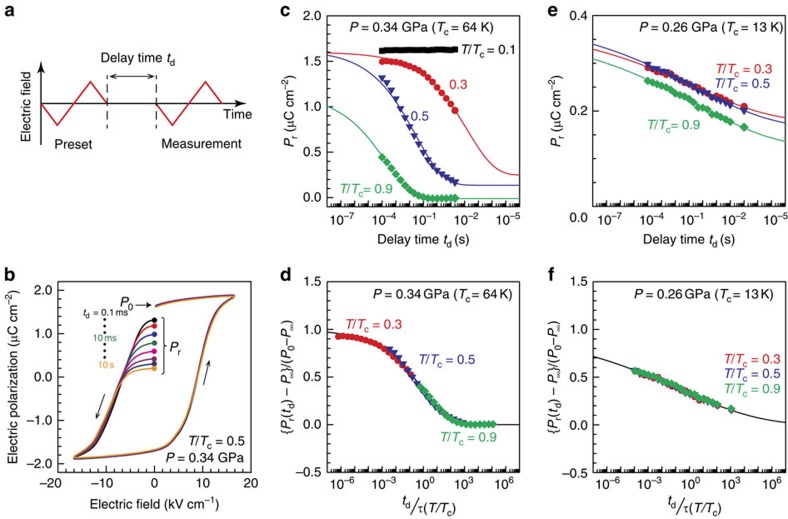
Polarization-decay properties under the influence of thermal and quantum fluctuations. (**a**) The measurement protocol for probing the polarization decay. (**b**) Typical polarization hysteresis loops with various delay times, measured at 0.34 GPa and *T*/*T*_c_=0.5. (**c**) The delay-time dependences of the remnant polarization at select temperatures and 0.34 GPa (*T*_c_≈64 K). (**d**) The normalized relaxation behaviour of the polarization decay at 0.34 GPa. (**e**) The delay-time dependence of the remnant polarization at 0.26 GPa (*T*_c_≈13 K), which is near the ferroelectric QCP. (**f**) The normalized relaxation behaviour of the polarization decay at 0.26 GPa. The lines in **c**–**f** represent fits to the standard relaxation equation; see equation 1.

**Figure 4:**
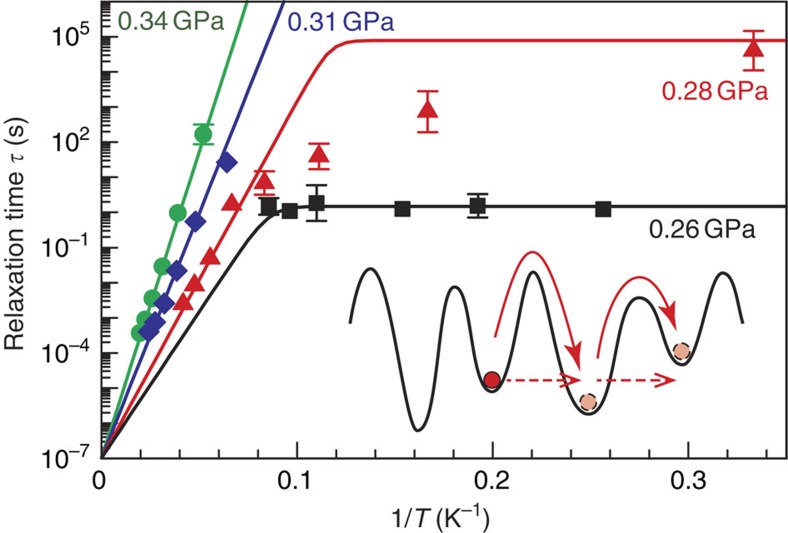
Thermal–athermal crossover of the domain-wall dynamics. The lines represent fits to Matthiessen's rule: 1/*τ*=1/*τ*_classical_+1/*τ*_quantum_, where *τ*_quantum_ is constant at a given pressure and *τ*_classical_ follows the Arrhenius law. The appreciable deviation of the simplest Matthiessen's rule from the experimental data at 0.28 GPa is partly attributable to the pressure inhomogeneity that arises from the pressure medium and the sample imperfection. The error bars represent the numerical ambiguity of *τ* obtained from the fitting results in Fig. 3. The inset is a schematic diagram comparing the classical thermally activated creep (solid arrows) with the quantum athermal creep (broken arrows) of a domain wall (modelled as a particle). The multi-valley schematic represents a multidimensional potential landscape.

Athermal domain-wall creep invokes successive quantum tunnelling between adjacent minima in the multidimensional potential landscape (Fig. 4, inset). In general, quantum tunnelling within an isolated double-well-potential system has been empirically verified by observing the crossover behaviour of the relaxation-type dynamics, in response to the development of quantum fluctuations, from the Arrhenius-type (classical) regime to the temperature-invariant (quantum) regime^20^,^21^,^22^. However, because creep dynamics are inherently a form of kinetics that is distinct from relaxation behaviour^23^,^24^, it is not obvious how to define the relaxation time associated with creep dynamics. In this study, we focus on a relaxation phenomenon originating from the creep motions of ferroelectric domain walls, that is, the decay of the net macroscopic polarization^25^,^26^. In realistic ferroelectrics, the remnant polarization after polarization switching may decay with time as a result of the nucleation of domains with the opposite polarization and their subsequent growth via domain-wall creep; in fact, such polarization decay towards a multidomain state has been observed in TTF-QCl_4_ (or TTF-CA), which is composed of stacking of TTF and QCl_4_ similarly to the case of TTF-QBr_2_I_2_ (ref. 27). As will be discussed below (Fig. 3d and Supplementary Fig. 3), we find that the polarization decay in TTF-QBr_2_I_2_ can, in fact, be described within the framework of relaxation behaviour, allowing us to extract the relaxation time associated with the creep dynamics and, thus, discuss the classical-to-quantum crossover.

We measured two successive polarization hysteresis loops with a delay time of *t*_d_, following the electric-field protocol illustrated in Fig. 3a (for details, see the Methods section) and found that the remnant net polarization, *P*_r_, appreciably decayed with time (Fig. 3b). The observed *t*_d_ dependences of *P*_r_ at various values of *T*/*T*_c_ under 0.34 GPa are summarized in Fig. 3c. As expected from the thermal domain-wall creep, dramatic prolongation of the polarization retention was observed towards low temperatures, and at *T*/*T*_c_=0.1, no polarization decay was discernible on the considered timescale (1 × 10^–4^–3 × 10^1^ s). In extracting the relaxation time *τ* from the decay profile, we exploited the standard relaxation equation:


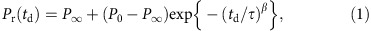


where *P*_0_ and *P*_∞_ denote the values of *P*_r_ in the limits of *t*_d_→0 (indicated in Fig. 3b) and *t*_d_→∞, respectively, and *β* is the phenomenological stretching parameter that represents the distribution of *τ*. We assumed that the value of *β* does not depend on temperature to avoid fitting ambiguity because of the limited *t*_d_ range of the data set (for the pressure dependence of *β*, see Supplementary Fig. 4); in fact, when {*P*_r_(*t*_d_)−*P*_∞_}/(*P*_0_−*P*_∞_) is plotted as a function of the normalized time, *t*_d_/*τ*, for each *T*/*T*_c_, the polarization-decay profiles yield a universal relaxation curve (Fig. 3d). The fitting using equation 1 is also successful for the data at 0.31 and 0.28 GPa (Supplementary Fig. 3), thereby validating the application of the relaxation equation to the polarization-decay properties. The results at 0.26 GPa (Fig. 3e) were also analysed (Fig. 3f), but in this case, the relaxation process is nearly independent of temperature, corroborating the athermal nature of the domain-wall creep at 0.26 GPa. In this way, the *τ*−1/*T* profiles were derived at various pressure values, that is, at varying proximity to the QCP (at 0.25–0.26 GPa), as shown in Fig. 4.

### Classical-to-quantum crossover

In Fig. 4, three key observations can be highlighted: first, whereas the *τ*−1/*T* profile exhibits Arrhenius-type behaviour at 0.34 and 0.31 GPa, it is temperature-invariant within the experimental accuracy at 0.26 GPa; second, at an intermediate pressure of 0.28 GPa, the crossover behaviour from the Arrhenius regime to the temperature-invariant regime is evident; and third, the saturated *τ* value in the temperature-invariant regime, *τ*_quantum_, is smaller at 0.26 GPa (≈*p*_c_) than at 0.28 GPa. The thermal-to-athermal crossover of *τ* towards the QCP is consistent with the empirical evidence used to validate quantum tunnelling^20^,^21^,^22^, thus leading us to conclude that near the ferroelectric QCP, the domain-wall creep is facilitated exclusively by successive quantum tunnelling rather than by thermal activation.

## Discussion

Finally, to quantitatively evaluate the validity of this quantum-mechanical domain-wall creep, we estimate the effective mass of the ferroelectric domain wall, *m*_eff_, using the simplest Wentzel–Kramers–Brillouin approximation^21^,^22^:


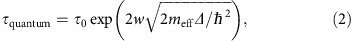


where *w* is the domain-wall tunnelling distance (where we adopt a value of≈7.29 Å (6.5 K, 0.2 GPa) as the unit-cell length along the stacking direction), 2π*τ*_0_ is the inverse of the attempt frequency, and *Δ* represents the activation barrier relevant to the Arrhenius behaviour. The values of the three quantities *τ*_quantum_, *τ*_0_, and *Δ* are available from the data at 0.28 GPa (for the details of the analysis, see Supplementary Note 2); from these values, the order of magnitude of *m*_eff_ at 0.28 GPa is estimated to be ≈6–8 × 10^2^ *m*_e_, where *m*_e_ denotes the electron mass. Given that the proton, which is anticipated to be capable of quantum tunnelling in solids^28^,^29^, has a mass of ≈1.8 × 10^3^ *m*_e_, the estimated *m*_eff_ is reasonably small. Moreover, *m*_eff_ is much smaller than the masses of the TTF and QBr_2_I_2_ molecules (≈3.7 × 10^5^ and ≈9.4 × 10^5^ *m*_e_, respectively), although the domain-wall creep entails the displacement of TTF and QBr_2_I_2_ molecules (Fig. 1b; see also Supplementary Fig. 1). A similar situation can also be observed in polyacetylene, in which the mass of the building blocks (carbon atoms) is ≈2.2 × 10^4^ *m*_e_, whereas the *m*_eff_ of the bond soliton (or, equivalently, a misfit in the C=C paring) is much lighter, only≈10 *m*_e_ (ref. 30). The small *m*_eff_ of the bond soliton in this material has been rationalized in terms of a large soliton width of ≈14 unit cells^31^, and it can therefore be expected that in TTF-QBr_2_I_2_, the domain-wall width should further broaden as quantum fluctuations develop; in fact, although the amount of quenched randomness is understood to be preserved upon a change in pressure, *Δ* decreases towards the ferroelectric QCP (Fig. 4 and Supplementary Fig. 5), suggesting an increase in the domain-wall width. We expect that such a broadened domain-wall width underlies the unexpectedly small effective mass and, thus, leads to the quantum-particle nature of the domain wall. The direct detection of the domain-wall width and domain structure^32^ is currently not possible, and such an effort would be challenging because the experiments would need to be performed under pressure; nevertheless, our observations establish that even a ferroelectric domain wall composed of heavy building blocks such as molecules can acquire a quantum-particle nature when a system is located near a ferroelectric QCP.

Recently, the development of permittivity towards a ferroelectric QCP has been drawing renewed attention and being extensively studied over a wide region of the paraelectric phase^33^,^34^. In the present study, by contrast, we establish that domain-wall motions, which normally become frozen and thus negligible at low temperatures, can affect the macroscopic physical properties in a ferroelectric phase even at the lowest temperature when quantum fluctuations are developing.

## Methods

### Sample preparation

The purchased TTF was purified via repeated recrystallization and vacuum sublimation. The QBr_2_I_2_ was synthesized and purified according to methods described in the literature^17^. Slow evaporation of a mixed acetonitrile solution of equimolar amounts of TTF and QBr_2_I_2_ under a stream of argon gas afforded 1:1 neutral TTF-QBr_2_I_2_ complex crystals as the minor product. Elongated plates of TTF-QBr_2_I_2_ were selectively grown at 0 °C in a second evaporation after seeding with a few crystals. The single crystals obtained have a typical dimension of 0.3 × 0.3 × 1.5 mm^3^.

### Permittivity and polarization measurements under pressure

A clamp-type dual-structure CuBe-NiCrAl cylinder cell was used for the pressure measurements. Daphne 7373 oil was used as the pressure-transmitting medium. Pressure inhomogeneity is likely present as inferred from the pressure dependence of β (Supplementary Fig. 4). The permittivity was measured along the *b* axis (the stacking direction) at 1 kHz, unless otherwise specified, by using an LCR meter (Agilent Technologies, E4980A). To remove the background contributions from the parasitic capacitance (≈0.1 pF), which is approximately one-tenth of the sample capacitance at the lowest temperature at 0.34 GPa (Fig. 2a), the open compensation was performed before the permittivity measurements on the sample. The polarization hysteresis loops (Fig. 1d, inset, and Fig. 3b) were measured at 10 kHz by using a commercial ferroelectric tester (Radiant Technologies, Precision Premier II). The measurements were performed for three single crystals, and we confirmed the reproducibility.

### Polarization-retention measurements

The polarization-retention properties were measured according to the electric-field protocol illustrated in Fig. 3a; during the preset process, a triangular electric pulse larger than the coercive electric field was applied to prepare a nearly monodomain ferroelectric state, and then, after a delay time *t*_d_, a measurement pulse of the same magnitude was applied to probe to what extent the sample remained polarized as a function of *t*_d_.

## Additional information

**How to cite this article:** Kagawa, F. *et al*. Athermal domain-wall creep near a ferroelectric quantum critical point. *Nat. Commun.* 7:10675 doi: 10.1038/ncomms10675 (2016).

## Supplementary Material

Supplementary Information Supplementary Figures 1-5, Supplementary Notes 1-2
