# Supplementary material for: Athermal domain-wall creep near a ferroelectric quantum critical point
Source: Nat Commun. 2016 Feb 16;7:10675. doi: 10.1038/ncomms10675 (PMC4757756; doi:10.1038/ncomms10675)
Supplement: Supplementary Information — Supplementary Figures 1-5, Supplementary Notes 1-2 [file ncomms10675-s1.pdf]

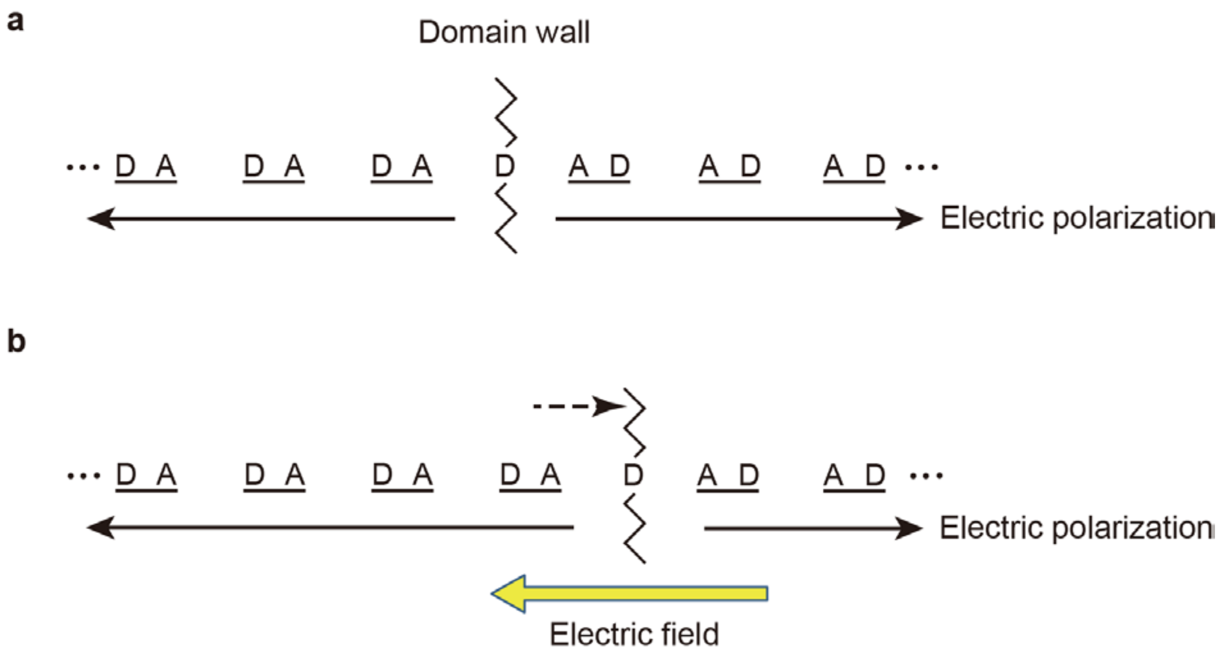

Supplementary Figure 1 | Schematic diagrams of the domain-wall structure and the domain-wall displacement under an electric field. a, The structure in the case of a tail-to-tail domain wall. **b,** The shift of the domain wall under an applied electric field. TTF and QBr_2I_2 are denoted by D (donor) and A (acceptor), respectively, and the underlines represent the dimerization of the two molecules. For simplicity, the domain wall is depicted as an atomically thin boundary, but in reality, it likely has a finite width, particularly when the system is located near the ferroelectric quantum critical point (for details, see the main text).

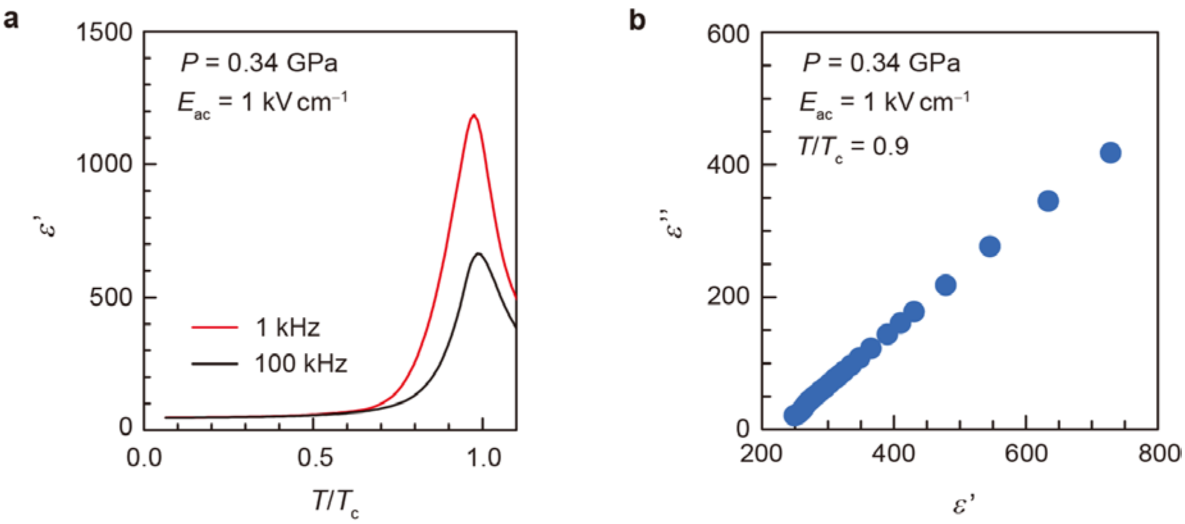

Supplementary Figure 2 | Frequency dependence of the permittivity at 0.34 GPa. **a,** The ε'-temperature profiles measured at 1 and 100 kHz. **b,** The Cole-Cole representation at T/T_c = 0.9. ε' and ε'' denote the real and imaginary parts, respectively, of the permittivity. In **b**, ε' and ε'' exhibit a linear relationship, consistently with the recent understanding of the permittivity originating from domain-wall creep motions (see ref. 24 in the main text). Error bars are smaller than the line width or symbol.

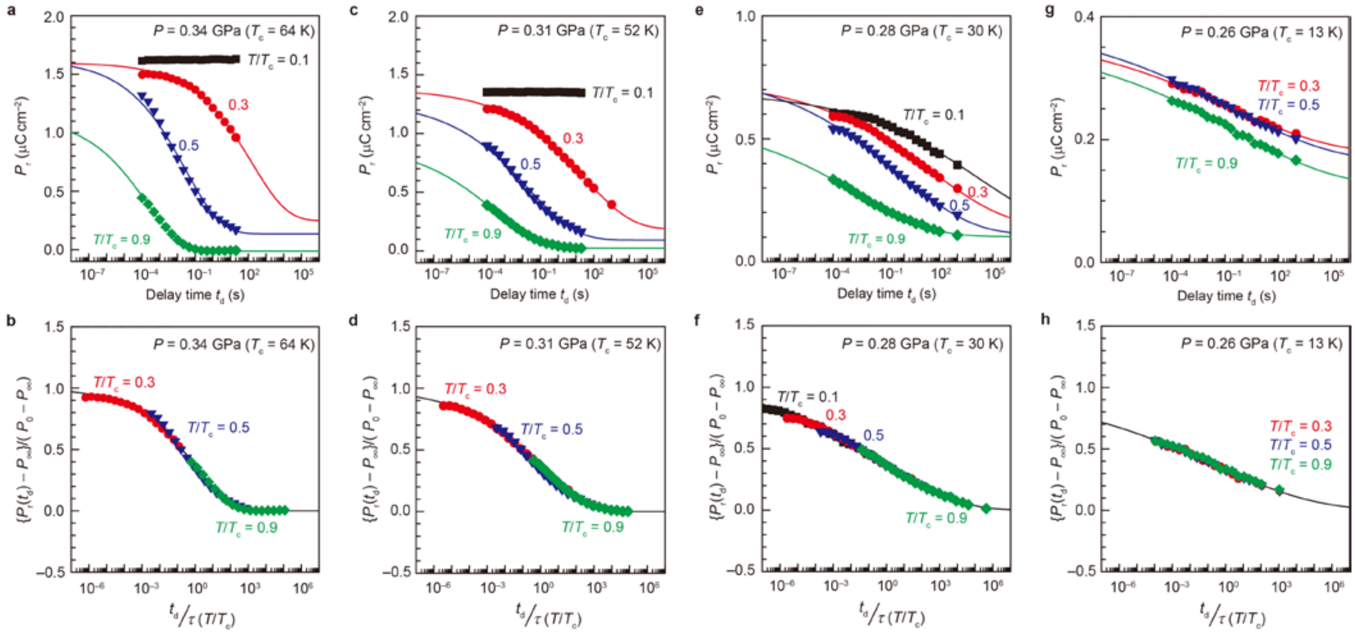

Supplementary Figure 3 | Polarization-decay properties under the influence of thermal and quantum fluctuations. a,c,e,g, The delay-time dependences of the remnant polarization at select temperatures and 0.34 GPa (**a**), 0.31 GPa (**c**), 0.28 GPa (**e**), and 0.26 GPa (**g**). **b,d,f,h,** The normalized relaxation behaviour of the polarization decay at 0.34 GPa (**b**), 0.31 GPa (**d**), 0.28 GPa (**f**), and 0.26 GPa (**h**). The lines in **a**-**h** represent fits to the standard relaxation equation; see Eq. (1) in the main text. **a,b,g,h** are the same with those shown in the main text.

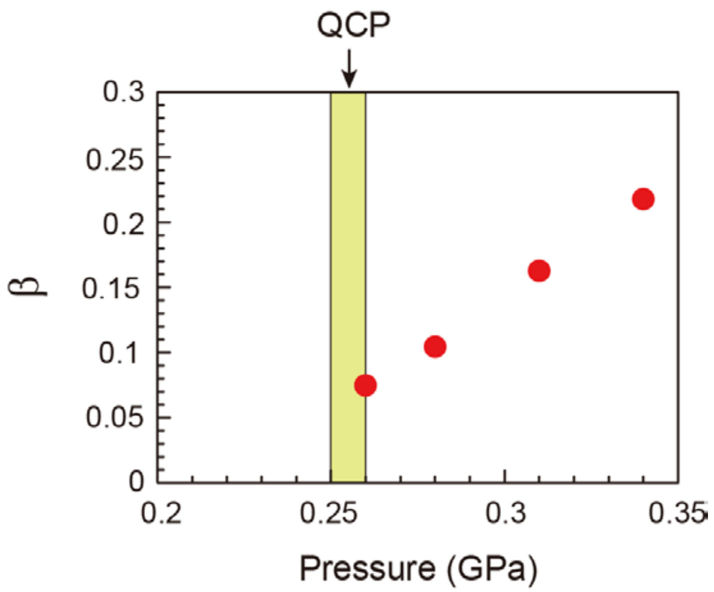

Supplementary Figure 4 | Pressure dependence of β. The vertical yellow bar represents the critical pressure of the quantum critical point (QCP). For the definition of β, see Eq. (1) in the main text. One can see that as the system approaches the QCP, the value of β decreases or, equivalently, the distribution of τ becomes more pronounced. This tendency is well accounted for by considering the presence of the pressure inhomogeneity that inevitably arises from the pressure media (solidified Daphne 7373) and the sample imperfection. Because T_c changes steeply as a function of pressure near the QCP (Fig. 1c in the main text), the consequence of the pressure inhomogeneity becomes increasingly pronounced toward the QCP.

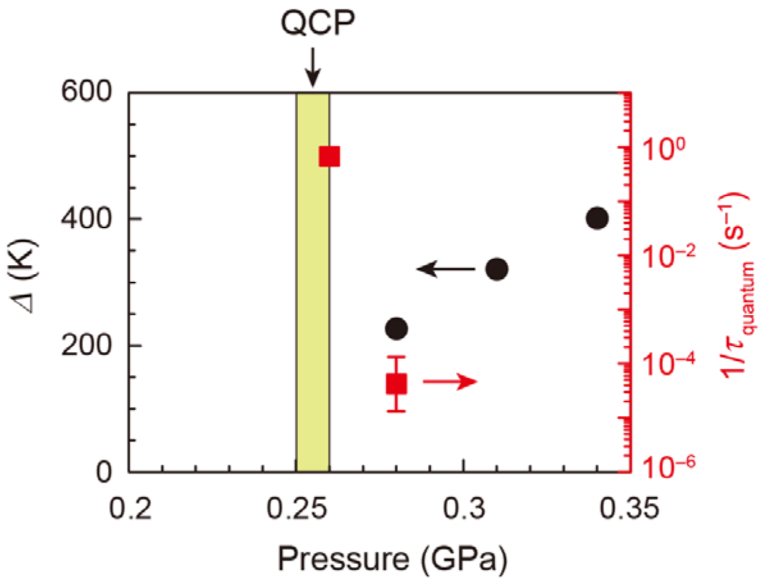

Supplementary Figure 5 | Pressure dependence of Δ and $1/\tau_{\mathrm{quantum}}$. These data were derived from Fig. 4 (the main text) in the framework of Matthiessen's rule, $1/\tau = 1/\tau_{\mathrm{classical}} + 1/\tau_{\mathrm{quantum}}$, where τ_{quantum} is constant at a given pressure and $\tau_{\mathrm{classical}}$ follows the Arrhenius law, $\tau_{\mathrm{classical}} = \tau_0 \exp(\Delta/k_B T)$ ($2\pi\tau_0$ is the inverse of the attempt frequency, Δ represents the activation barrier, and k_B is Boltzmann's constant). The error bar represents the ambiguity in estimating τ_{quantum} in Fig. 4 and corresponds to the uncertainty of the estimated domain-wall mass, 6-8×10^2 m_e.

Supplementary Note 1: Slight difference in the peak temperature in Fig. 2a.

In Fig. 2a in the main text, the peak temperature is slightly lower for a higher a.c. electric field. The origin has not been exclusively identified at present. A plausible scenario is that the dielectric contribution from the domain-wall creep takes its maximum slightly below the transition temperature because the domain and domain wall are innately not discernible at T_c because of the divergence of the correlation length; as a result, the dielectric response due to the domain-wall creep is expected to become well defined at temperatures lower than T_c.

Supplementary Note 2: Estimation of the quantum-tunneling dynamics

To estimate the quantum-tunneling dynamics in the temperature-invariant regime (Fig. 4 in the main text), we characterized the overall behaviour of τ by using the simplest form of Matthiessen's rule: $1/\tau = 1/\tau_{\mathrm{classical}} + 1/\tau_{\mathrm{quantum}}$, where τ_{quantum} is constant at a given pressure and $\tau_{\mathrm{classical}}$ follows the Arrhenius law, $\tau_{\mathrm{classical}} = \tau_0 \exp(\Delta/k_B T)$ ($2\pi\tau_0$ is the inverse of the attempt frequency, Δ represents the activation barrier, and k_B is Boltzmann's constant). In fitting the results, we assumed τ_0 to be pressure-independent. The fitted curves are displayed in Fig. 4 of the main text. Thus, the pressure dependence of Δ and $1/\tau_{\mathrm{quantum}}$ could be derived, as shown in Supplementary Fig. 5. As the pressure approaches p_c ($\approx$ 0.25-0.26 GPa) from above, Δ decreases. This behaviour partially accounts for the increase in the quantum relaxation rate, $1/\tau_{\mathrm{quantum}}$, as indicated by Eq. (2) in the main text.
